# Model-based cost-effectiveness analysis of oral antivirals against SARS-CoV-2 in Korea

**DOI:** 10.4178/epih.e2022034

**Published:** 2022-03-12

**Authors:** Youngji Jo, Sun Bean Kim, Munkhzul Radnaabaatar, Kyungmin Huh, Jin-Hong Yoo, Kyong Ran Peck, Hojun Park, Jaehun Jung

**Affiliations:** 1 Section of Infectious Disease, Department of Medicine, Boston Medical Center, Boston, MA, USA; 2 Department of Internal Medicine, Division of Infectious Diseases, Korea University College of Medicine, Seoul, Korea; 3 Artificial Intelligence and Big-Data Convergence Center, Gil Medical Center, Gachon University College of Medicine, Incheon, Korea; 4 Division of Infectious Diseases, Department of Medicine, Samsung Medical Center, Sungkyunkwan University School of Medicine, Seoul, Korea; 5 Division of Infectious Diseases, Department of Internal Medicine, Bucheon St. Mary’s Hospital, Bucheon, Korea; 6 Division of Infectious Diseases, Department of Internal Medicine, Bucheon St. Mary’s Hospital, Bucheon, Korea; 7 Prime Minister’s Secretariat, Seoul, Korea

**Keywords:** SARS-CoV-2, Cost-effectiveness analysis, Antiviral agents, COVID-19, Hospitalization

## Abstract

**OBJECTIVES:**

Many countries have authorized the emergency use of oral antiviral agents for patients with mild-to-moderate cases of coronavirus disease 2019 (COVID-19). We assessed the cost-effectiveness of these agents for reducing the number of severe COVID-19 cases and the burden on Korea’s medical system.

**METHODS:**

Using an existing model, we estimated the number of people who would require hospital/intensive care unit (ICU) admission in Korea in 2022. The treatment scenarios included (1) all adult patients, (2) elderly patients only, and (3) adult patients with underlying diseases only, compared to standard care. Based on the current health system capacity, we calculated the incremental costs per severe case averted and hospital admission for each scenario.

**RESULTS:**

We estimated that 236,510 COVID-19 patients would require hospital/ICU admission in 2022 with standard care only. Nirmatrelvir/ritonavir (87% efficacy) was predicted to reduce this number by 80%, 24%, and 17% when targeting all adults, adults with underlying diseases, and elderly patients (25, 8, and 4%, respectively, for molnupiravir, with 30% efficacy). Nirmatrelvir/ritonavir use is likely to be cost-effective, with predicted costs of US$8,878, US$8,964, and US$1,454, per severe patient averted for the target groups listed above, respectively, while molnupiravir is likely to be less cost-effective, with costs of US$28,492, US$29,575, and US$7,915, respectively.

**CONCLUSIONS:**

In Korea, oral treatment using nirmatrelvir/ritonavir for symptomatic COVID-19 patients targeting elderly patients would be highly cost-effective and would substantially reduce the demand for hospital admission to below the capacity of the health system if targeted to all adult patients instead of standard care.

## GRAPHICAL ABSTRACT

[Fig f3-epih-44-e2022034]

## INTRODUCTION

Since the World Health Organization (WHO) declared the novel coronavirus disease 2019 (COVID-19) outbreak a pandemic on March 11, 2020, various therapeutics have been tested to treat or prevent COVID-19 worldwide. The U.S. Food and Drug Administration recently authorized Pfizer’s nirmatrelvir and ritonavir and Merck’s molnupiravir for emergency use in the treatment of mild-to-moderate COVID-19 in adults who tested positive for direct severe acute respiratory syndrome-coronavirus-2 (SARS-CoV-2) and those with a high-risk of developing severe COVID-19 leading to possible hospitalization or death [1,2]. In clinical trials, these oral antiviral agents reduced the risk of hospitalization or all-cause mortality over a 29-day follow-up period among individuals with chronic medical conditions or an increased risk of SARS-CoV-2 infection and those who had not received a COVID-19 vaccine for other reasons [3,4]. Unlike other effective therapeutics, including remdesivir, dexamethasone, some immunomodulators, and SARS-CoV-2 monoclonal antibodies, these novel agents are readily accessible in orally bioavailable form at home. However, these novel agents are expensive and their initial supply is too low to treat all individuals infected with SARS-CoV-2, most of whom have mild-to-moderate symptoms; approximately 81% of ongoing SARS-CoV-2 cases are reportedly mild to moderate, while 14% are severe and 5% are critical [5-7]. Therefore, the use of these antivirals should be prioritized for high-risk patients to achieve the best public health and treatment outcomes. Moreover, the effectiveness of these treatments in the real-world could look different from the results of well-designed clinical trials; therefore, various factors must be considered. Since phase 3 clinical trials of oral antiviral agents were conducted with nonvaccinated patients, there is some uncertainty concerning their estimated effect on vaccinated patients at this point. This study aimed to examine the cost-effectiveness of using novel oral antiviral agents to decrease the number of patients infected with severe COVID-19 and reduce the burden of SARS-CoV-2 on the medical system in terms of hospital/intensive care unit (ICU) admissions and treatment costs based on the current capacity of the health system.

## MATERIALS AND METHODS

In Korea, there are an estimated 25,000 general hospital beds and 1,500 ICU beds available for COVID-19 patients as of January 2022. Based on the existing COVID-19 epidemiology model (Supplementary Materials 1 and 2), we projected the peak number of hospital/ICU beds based on hospital/ICU prevalence and the number of people who would require hospital/ICU admission based on the incidence of hospital/ICU admission in Korea between January and December 2022. We assumed that the use of treatments (molnupiravir or nirmatrelvir/ritonavir) would reduce the admission rate based on the results of recent clinical trials, with a 30% efficacy rate for molnupiravir and an 87% efficacy rate for nirmatrelvir/ritonavir compared to standard care [3,4]. We also assumed these treatments would reduce the recovery time in the hospital/ICU by 4 days (from an average of 13 to 9 days), thus allowing more patients to be admitted during months when the hospital/ICU capacity is exceeded [8]. Since there were no data estimating the decrease in the duration of patients’ hospital/ICU stays as a result of taking oral antiviral agents in clinical studies, we assumed a median reduced length of stay of 4 days as a result of taking oral antiviral agents based on a range from 1 day to 8 days according to the results of other studies on antiviral agents, including on the effects of remdesivir on COVID-19 and oseltamivir on influenza [9-12]. We used estimates of the peak number of hospital/ICU beds (by month) to identify months with peak capacity (“1” if hospital/ICU capacity was exceeded; “0” if hospital/ICU capacity was not exceeded). We then used the months with peak capacity and the total number of people who required hospital/ICU admission to identify the total number of likely hospital/ICU admissions during peak and non-peak months (same as the original estimate of the number of people who require hospital/ICU admission) (Supplementary Material 3). Using Korean COVID-19 hospitalization data and treatment guidelines, we estimated COVID-19-related hospital/ICU admissions and treatment costs for 3 targeted risk groups: (1) all adult patients (aged 20 years and older), (2) elderly patients (aged 60 years and older), and (3) adult patients with underlying diseases, all of which would be treated with molnupiravir or nirmatrelvir/ritonavir compared to standard care (without molnupiravir or nirmatrelvir/ritonavir). Based on the empirical data and published literature, we accounted for age and condition-specific (i.e., underlying disease-related) progression rates and hospital admission rates based on data from a non-publicly available Korea Disease Control and Prevention Agency database (Table 1) [13].

Based on the previously published literature, we assumed that 33% of patients who tested positive would have underlying diseases. One course of molnupiravir or nirmatrelvir/ritonavir (oral administration) was assumed to be 40 pills and 30 pills, respectively, over 5 days (Table 1). Our primary outcome measures were the number of prevented severe cases (cases that required hospital/ICU admission) and the net COVID-19-related hospital/ICU admissions or prevented admissions based on the efficacy of treatment for reducing the hospital admission rate and the inpatient recovery time according to the treatment scenarios for the respective target risk groups. In our study, the criteria for severity were cases that required hospital, general ward, or ICU admission in which the patient has an oxygen saturation level (SpO_2_) of < 94% in a room at sea level, a ratio of arterial partial pressure of oxygen to fraction of inspired oxygen (PaO_2_/FiO_2_) of < 300 mmHg, a respiratory rate of > 30 breaths/min, or lung infiltration of > 50% based on radiological imaging according to the severity of illness categories of the National Institute of Health [14].

We estimated the resource use for each scenario from the perspective of the Korean health system for all hospital/ICU person-days accrued by COVID-19 patients between January and December 2022. We estimated the costs associated with 3 metrics: (1) the cost of monthly treatment for the health system during peak and non-peak months, (2) the cost of monthly treatment for the health system without oral antiviral treatment (status quo) during peak and non-peak months, and (3) the total treatment cost. During peak months, the health system costs were calculated based on the total number of admissions (83,333 patients in hospitals and 5,000 patients in ICUs per month with treatment caused by treatment’s recovery time benefits; 57,692 patients in hospitals and 3,462 patients in ICUs per month without treatment) multiplied by the hospital/ICU cost per person. During non-peak months, the health systems costs were calculated based on the total number of admissions (the number of patients requiring hospital/ICU admission) multiplied by the hospital/ICU cost per person. We used an average hospital cost of US$267 (range, 136 to 452) per person per day and an average ICU cost of US$825 (range, 550 to 1,100) per person per day based on data from Central Disaster and Safety Countermeasure Headquarters [18]. We assumed the additional cost for a full course of molnupiravir or nirmatrelvir/ritonavir (US$700 per patient course) for each corresponding scenario (Table 2).

The costs were converted from Korean won to US dollars based on the December 2021 average exchange (1 US dollar=1,100 Korean won) [19]. The incremental cost-effectiveness was calculated (as the incremental cost per prevented severe case and incremental cost per net admission) in 2021 US dollars for each therapeutic scenario across the risk groups compared to standard care. We assessed a wide range of possible treatment efficacy rates (10-90%) for reducing the disease severity rate based on real-world efficacy scenarios such as the timing of drug intake with imperfect diagnostic sensitivity, treatment adherence with potential toxicity/side effects, the relative treatment efficacy according to vaccination status, and patient age or other comorbidities. We also performed 1-way sensitivity analyses of the key model parameters to determine the robustness of the primary results, expressed as the incremental cost per prevented severe case, to determine the uncertainty of the individual model parameters.

### Ethics statement

This study was approved by the Institutional Review Board of Gachon University College of Medicine, Incheon, Korea (IRB No. GFIRB2021-232), and participant consent was waived by the ethics committee since the study examined routinely collected medical data that were processed anonymously at all stages. The study was conducted ethically according to the World Medical Association’s Declaration of Helsinki.

## RESULTS

The estimated total number of severe COVID-19 cases requiring hospital/ICU admission in Korea from January to December 2022 was 236,510 without treatment using molnupiravir or nirmatrelvir/ritonavir (standard care); 48,032 with treatment using nirmatrelvir/ritonavir targeting all adult patients; 95,658 with treatment using nirmatrelvir/ritonavir targeting elderly patients only; 179,963 with treatment using nirmatrelvir/ritonavir targeting adult patients with underlying diseases only; 176,543 with treatment using molnupiravir targeting all adult patients; 226,871 with treatment using molnupiravir targeting elderly patients only; and 218,513 with treatment using molnupiravir targeting adults with underlying diseases only. Overall, nirmatrelvir/ritonavir, at 87% efficacy, was estimated to reduce the number of severe COVID-19 cases requiring hospital/ICU admission by 80%, 24%, and 17% when targeting all adult patients, adult patients with underlying diseases only, and elderly patients only, respectively, compared to 25%, 8%, and 4% reductions resulting from treatment with molnupiravir, which has an efficacy rate of 30% (Table 2). Under the current health system capacity, hospital and ICU capacity are expected to be exceeded for 2-4 months, respectively, with standard treatment, with an anticipated 204,580 admissions. However, the number of months during which capacity is exceeded will decrease to 0-4 months depending on treatment efficacy and target groups, with a predicted total of 51,029 admissions resulting from treatment with nirmatrelvir/ritonavir targeting all adult patients; 197,159 admissions resulting from treatment with nirmatrelvir/ritonavir targeting elderly patients only; 163,346 admissions resulting from treatment with nirmatrelvir/ritonavir targeting adults with underlying diseases only, 160,575 admissions resulting from treatment with molnupiravir targeting all adult patients, 259,363 admissions resulting from treatment with molnupiravir targeting elderly patients only, and 256,112 admissions resulting from treatment with molnupiravir targeting adult patients with underlying diseases only. These decreases are the results of the combined efficacy of treatment for reducing hospital admission rates and recovery time within the corresponding target populations (Supplementary Material 4).

Treatment efficacy and future epidemic scenarios together influence incremental costs and the demand for hospital/ICU admission. The number of prevented severe cases was the highest when treatment targeted all adult patients, at 188,478 cases with treatment using nirmatrelvir/ritonavir (ranging from 19,356 to 205,442 cases corresponding to efficacy rates spanning 10-90%), followed by when treatment targeted patients with underlying diseases only, at 56,547 cases (ranging from 5,806 to 61,634 cases depending on the efficacy rate), and when treatment targeted elderly patients only, at 40,852 cases (ranging from 2,847 to 46,448 according to the efficacy rate) (Table 2 and Supplementary Material 4). The impact on reducing the total number of severe cases was estimated to be lower when treatment targeted elderly patients only or adult patients with underlying diseases only given the smaller size of the target populations compared to treatment targeting all adult patients, but more patients could be admitted due to the recovery time benefit during months when the hospital/ICU capacity is exceeded. If the efficacy for reducing the admission rate is higher than 30%, treatment targeting all adult patients can suppress the epidemic curve below the hospital capacity limit for all months and prevent nearly 44,005 admissions at 30% efficacy and 173,512 admissions at 90% efficacy (Table 2 and Supplementary Material 4). If treatment reduces the admission rate by 10%, but still reduces the inpatient recovery time to 4 days during months when the hospital/ICU capacity is exceeded, treatment for all adult patients would result in an additional 51,051 admissions.

**Figure 1.**
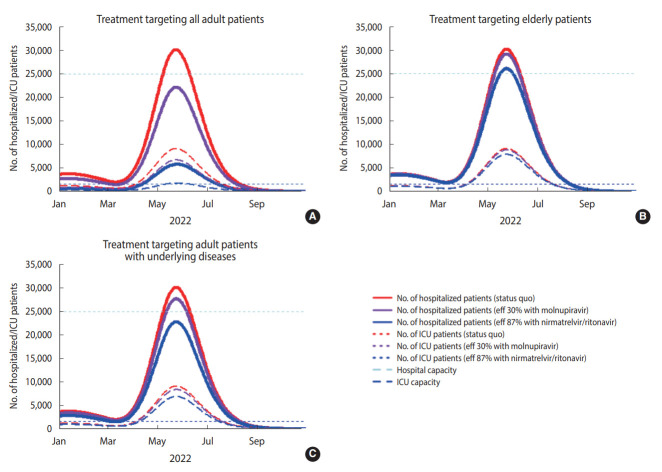
Number of hospitalized COVID-19 infected patients for each treatment scenario (standard care, molnupiravir, and nirmatrelvir/ritonavir). Treatment targeting all adult patients (A) elderly patients (B), and adults patients with underlying diseases (C). eff, efficacy; ICU, intensive care unit.

The total operating costs of COVID-19-related hospital/ICU care for the health system in Korea between January and December 2022, excluding the cost of drugs, would be US$66 million with standard care. The administration of nirmatrelvir/ritonavir targeting all adult patients, with an 87% efficacy rate for reducing hospital/ICU admission, may reduce the cost to US$21 million, as it could substantially reduce the total number of severe cases. However, the same strategy could increase the operating costs of the health system to US$67 million and US$58 million when targeting only elderly patients and only adult patients with underlying diseases, respectively. In terms of drug costs, we estimated that, in 2022, US$1.7 billion would be required to administer molnupiravir or nirmatrelvir/ritonavir targeting all adult patients, while US$58 million would be required to administer treatment targeting elderly patients only and US$515 million would be required to administer treatment targeting adult patients with underlying diseases only (Figure 1).

Collectively, if targeting elderly patients, all adult patients, and adults with underlying diseases, treatment with nirmatrelvir/ritonavir would result in cost-effective incremental ratios of US$1,454, US$8,878, and US$8,964, respectively, per prevented severe case. These strategies would result in an epidemic resurgence that overwhelms the current health system capacity when targeting elderly patients only but would suppress the epidemic curve enough to remain below the current health system capacity when targeting all adult patients and adult patients with underlying diseases. This translates to US$8,006, US$10,898, and US$12,293, respectively, for every additional hospital/ICU admission that is prevented. On the contrary, treatment with molnupiravir targeting elderly patients, all adult patients, and adults with underlying diseases would result in incremental cost-effectiveness ratios of US$7,915, US$28,492, and US$29,575, respectively, for every severe case that is prevented, which translates to US$1,393, US$38,828, and US$10,329 for every additional hospital/ICU admission. Varied treatment efficacy rates for reducing the admission rate from 10-90% would result in a decreased cost per prevented severe case ranging from US$89,617 to US$8,101 and US$26,994 to US$1,263 if the treatment targeted all adult patients and elderly patients only, respectively. If the real-world treatment efficacy remains high (> 87%), oral treatment of symptomatic COVID-19 patients can be generally cost-effective and reduce the demand for hospital admission across all target group scenarios (Supplementary Material 4).

**Figure 2.**
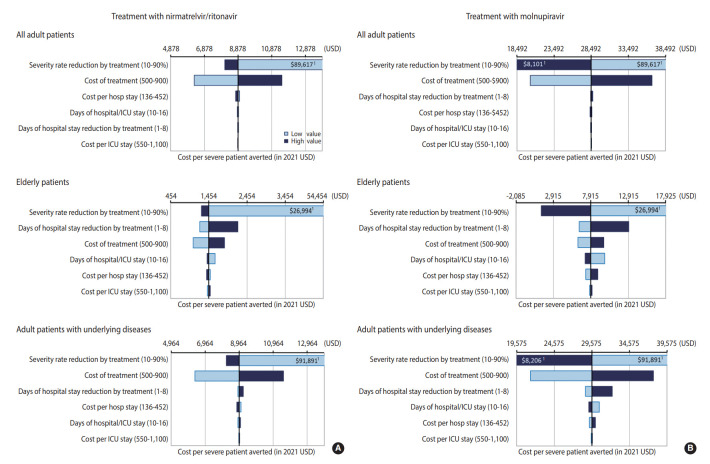
One-way sensitivity analyses in Korea, comparing between the incremental baseline cost and net admissions by risk group scenario (A) treatment with nirmatrelvir/ritonavir; (B) treatment with molnupiravir. A decrease in the length of hospital stay by treatment (higher recovery time benefit) is associated with an increased cost per prevented severe case (lower cost-effectiveness) since it allows more patients to be admitted during months when the capacity of the health system is exceeded, in turn increasing costs for the health system. USD, US dollar; ICU, intensive care unit. 1The bar goes beyond the range.

One-way sensitivity analysis revealed uncertainty concerning the cost per prevented severe case in each scenario for the risk groups compared to standard care. Under an expected high epidemic resurgence that could exceed the health system capacity, the treatment efficacy for reducing the severity rate was the key determinant of the incremental cost-effectiveness ratio associated with every scenario since it can substantially change the demand for hospital/ICU admission. If treatment targets a large population and has a high efficacy rate (e.g., if treatment with nirmatrelvir/ritonavir targets all adult patients or adult patients with underlying diseases only, which could reduce the total demand for hospital/ICU admission below the capacity of the health system), the cost of treatment would have the second-highest impact on the incremental cost-effectiveness ratio. However, if treatment targets a relatively small, high-risk population (e.g., if treatment with nirmatrelvir/ritonavir targets elderly patients, which may have a marginal impact on reducing the total demand for hospital/ICU admission), the reduction in the average length of patients’ hospital stays would have the second-highest impact on the incremental cost-effectiveness ratio with the benefit of reducing the average recovery time (Figure 2).

## DISCUSSION

Our study showed that the epidemic impact and cost-effectiveness of COVID-19 oral treatment may vary substantially depending on the magnitude of the real-world treatment efficacy for reducing the severity (or admission) rate and the size and characteristics of the target groups. In Korea, if a high epidemic resurgence is expected in 2022 that could exceed the capacity of the current health system, oral nirmatrelvir/ritonavir treatment for symptomatic COVID-19 patients can be considered highly cost-effective if targeted to elderly patients only (US$1,454 per prevented severe case) and can substantially reduce the demand for hospital/ICU admission (80%, 188,478 cases) to below the capacity of health system if targeted to all adult patients compared to these metrics with standard care. Given the capacity of the current health system, nirmatrelvir/ritonavir is moderately cost-effective, with costs of US$8,006, US$10,898, and US$12,293 for every hospital/ICU admission that is prevented if targeted to elderly patients only, all adult patients, and adult patients with underlying diseases only, respectively, compared to standard care.

Due to the consistent emergence of new SARS-CoV-2 variants, the burden of breakthrough infection or reinfection is growing despite increased vaccination uptake. Moreover, countries that have effectively controlled this unprecedented pandemic and have maintained a low prevalence rate are likely to experience a high epidemic resurgence in the future due to their large susceptible populations of COVID-19 infections. In this context, if treatment efficacy is as low as 10%, the treatment’s impact on the demand for hospital/ICU admission would be marginal, and a recovery time benefit that allows hospitals to receive additional patients would be the major value of oral antiviral treatment for all target group scenarios. In contrast, if treatment efficacy is as high as 90%, its impact on the demand for hospital/ICU admission would be substantial in all target groups, and it would be highly cost-effective, with costs of US$628, US$9,591, and US$11,169 for every hospital/ICU admission that is prevented by targeting elderly patients only, all adult patients, and adults with underlying diseases only, respectively. Targeting adult patients with underlying diseases only may achieve a similar incremental cost-effectiveness ratio but with only about 30% of the total costs (as well as health outcomes) of treatment targeting all adult patients.

Our study has some limitations. First, the size of the target population and treatment impact may be subject to future epidemic trends (e.g., the influence of new variants) and policy choices (e.g., social distancing policies). For example, the influence of the Omicron variant, which has a faster transmission rate [20,21] but a lower risk of severe illness than other variants [22-24], may reduce the cost-effectiveness of treatment since it is likely to increase the size of the target population and treatment costs but reduce the severity/admission rate and, thus, the demand for hospital/ICU admission. Second, we examined treatment efficacy based on reductions in severity/admission and did not account for the treatment efficacy according to the number of prevented deaths. If we consider prevented deaths, the value of treatment may be even higher, especially for high-risk patients such as seniors or adults with underlying diseases. Third, several factors may influence real-world treatment efficacy and cost-effectiveness. Studies have shown that treatment may be less effective when administered later in the course of the disease, particularly after patients have experienced symptoms for more than 5 days or after hospitalization [25]. Thus, early testing and monitoring are critical for achieving the maximum benefits of treatment and can further improve their cost-effectiveness. Fourth, our cost data did not include additional costs associated with drug prescriptions/administration and adverse events management, which may especially affect elderly patients or adult patients with underlying diseases. While our study was based on the highly cost-effective Korean medical system, if we consider these cost implications, the cost of treatment may be greater than the estimates in this study, and treatment may be less cost-effective in other countries with health systems that entail higher costs or where access is lower than in Korea. In addition, while an increasing number of patients within the capacity of a given hospital/ICU may achieve potential economies of scale, additional costs may also be required for medical facilities and medical personnel, especially if the demand for hospitalization nears or exceeds full capacity. While this may change the exact estimates in our results, it is unlikely to alter our conclusion that treatment is likely to have the greatest epidemic impact when targeting all adult patients but is most cost-effective when targeting elderly patients only. In addition, our study did not compare the 2 oral antiviral treatments but rather compared both antiviral treatments to standard care. A formal indirect treatment comparison was not performed, and the relative effectiveness and cost-effectiveness of each treatment should, therefore, not be inferred. Finally, in the clinical trials during which the oral therapeutics were tested, the end-point was measured as a composite outcome including both hospitalization and death. In our study, we took a more conservative approach by focusing such efficacy measures into reduced severity/admission rate. It is also because we considered death is a rather distant outcome measure and can be influenced by other recommended treatment regimes such as remdesivir, tocilizumab, dexamethasone to severely ill patients which is beyond our scope of analysis.

Nonetheless, this study has several strengths. First, to our knowledge, this is the first cost-effectiveness analysis of antiviral drugs based on real-world data and future trend prediction simulations from Korea. Our epidemiology model incorporated detailed disease progression and population dynamics such as age-specific contact patterns, testing performance, and vaccination uptake based on detailed empirical data that allowed us to estimate the relative total population impact by targeting specific risk groups (Supplementary Materials 1 and 2). Second, in addition to the overall population impact (i.e., decrease in the total number of severe cases by treatment), we considered the capacity of the health system to identify potential admission trade-offs according to the efficacy of each treatment, one of which would increase necessary admissions by reducing the recovery time benefit and the other one of which would decrease the demand for admission by reducing severity. This enabled a more accurate assessment of the implications for the health system concerning the demand for hospitalization and the cost of treatment related to each target scenario.

In conclusion, given the expected high epidemic resurgence in 2022 that could exceed the current capacity of Korea’s health system, oral nirmatrelvir/ritonavir treatment of symptomatic COVID-19 patients would be highly cost-effective if targeted to elderly patients (US$1,454 per prevented severe case) and could substantially reduce the demand for hospital/ICU admission (80%, 188,478 cases) below the capacity of the health system if targeted to all adult patients compared to standard care. The active introduction, prioritization, and administration of oral therapeutics can be a cost-effective strategy for reducing the burden of SARS-CoV-2 infection on medical systems, such as intensive care units when treating severe COVID-19 patients.

## Figures and Tables

**Figure f3-epih-44-e2022034:**
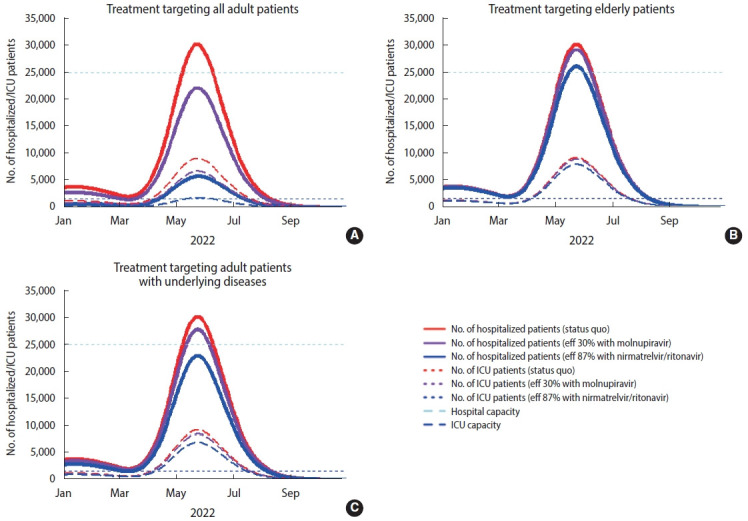


**Table 1.** Key input parameters

Health system and patient characteristics	Value			Reference
No. of hospital beds	25,000			KDCA
No. of ICU beds	1,500			KDCA
Percentage of test-positive patients with underlying diseases	33%			[15-17]
Probability of symptoms given infection, % (age, yr)	66 (0-19); 74 (20-39); 68 (40-59); and 62 (≥60)			KDCA
Hospital admission rate, % (age, yr)^1^	0.02 (0-19); 0.15 (20-39); 0.74 (40-59); and 7.96 (≥60)			KDCA
**Efficacy**	**Base**	**Low**	**High**	**Reference**
Average length of hospitalization for COVID-19 patients (day)	13	10	16	CDSCH
Hospital admission rate reduction from molnupiravir (%)	30	10	50	[3]
Hospital admission rate reduction from nirmatrelvir/ritonavir (%)	87	66	95	[4]
Reduced length of hospitalization from molnupiravir/nirmatrelvir/ritonavir (day)	4	1	6	Based on assumption
Reduced length of ICU stay from molnupiravir/nirmatrelvir/ritonavir (day)	4	1	8	Based on assumption
**Cost input (unit: USD)**	**Base**	**Low**	**High**	**Reference**
Health system operating cost per hospital bed day	267	136	452	KDCA
Health system operating cost per ICU bed day	825	550	1,100	KDCA
Cost of molnupiravir regimen (40 pills total across 5 days)	700	500	900	KDCA
Cost of nirmatrelvir/ritonavir regimen (30 pills total across 5 days)	700	500	900	CDSCH

KDCA, Korea Disease Control and Prevention Agency; ICU, intensive care unit; COVID-19, coronavirus disease 2019; CDSCH, Central Disaster and Safety Countermeasure Headquarters; USD, US dollar.

1 ICU admission rate was assumed to be 20% of hospital admission rate.

**Table 2.** Target populations, health outcomes, total costs, and ICERs of COVID-19 treatment scenarios in Korea in 2022

Variables		Standard care (without treatment)	Molnupiravir: 30% efficacy for reducing admission			Nirmatrelvir/ritonavir: 87% efficacy for reducing admission		
			All adult patients	Elderly patients only	Adult patients with underlying disease only	All adult patients	Elderly patients only	Adult patients with underlying disease only
Target population: Test-positive COVID-19 patients who reported symptoms within 5 days after diagnosis								
	No. of the target population		2,454,096	83,314	736,218	2,454,096	83,314	736,218
Health outcome by intervention scenario^1^								
	No. of severe patients who require hospital admission (A)^2^	181,931	135,803	174,517	168,088	36,949	150,506	138,433
	No. of severe patients who require ICU admission (B)	54,579	40,740	52,354	50,425	11,083	45,152	41,530
	Total no. of severe patients who require hospital/ICU admission (C)	236,510	176,543	226,871	218,513	48,032	195,658	179,963
	Total prevented severe cases (D)	NA	-59,967	-9,639	-17,997	-188,478	-40,852	-56,547
	No. of patients receiving hospital care during months when capacity is exceeded (E)	115,385	0^3^	166,667	166,667	0^3^	83,333	0^3^
	No. of patients receiving ICU care during months when capacity is exceeded (F)	13,846	20,000	20,000	20,000	10,000	20,000	20,000
	Hospital admission during months when capacity is not exceeded (G)^2^	68,873	135,803	66,380	63,480	36,949	88,009	138,433
	ICU admission during months when capacity is not exceeded (H)^4^	6,476	4,772	6,316	5,965	4,080	5,817	4,913
	Total admissions under the current health system capacity (I: E+F+G+H)	204,580	160,575	259,363	256,112	51,029	197,159	163,346
	Net total hospital/ICU admission by treatment under the current health system capacity (J)^5^	NA	-44,005	54,783	51,532	-153,551	-7,420	-41,234
Cost (million USD)								
	Drug costs (K)	NA	1,718	58	515	1,718	58	515
	Hospital costs (L)	49	36	62	61	10	46	37
	ICU costs (M)	17	20	22	21	12	21	21
	Total costs (N: K+L+M)	66	1,775	142	598	1,739	125	573
Incremental costs, million USD (O)		NA	1,709	76	532	1,673	59	507
ICER: Cost per prevented severe case, USD (D/O)		NA	28,492	7,915	29,575	8,878	1,454	8,964
ICER: Cost per admission/prevented admission, USD (J/O)^6^		NA	38,828	-1,393	-10,329	10,898	8,006	12,293

COVID-19, coronavirus disease 2019; ICER, incremental cost-effectiveness ratio; ICU, intensive care unit; Mol, molnupiravir; NA, not available; USD, US dollar.

1 The health outcome is the total population impact based on the epidemiology model targeting each respective patient group.

2 “Hospital admissions during months when capacity is not exceeded (G)” is the same as “Number of the population who require hospital admission (A)” for targeting all adult patients since the hospital capacity is never exceeded in all months of 2022.

3 0 since treatment targeting all adults patients can suppress the epidemic curve below the ICU capacity limit for all months.

4 Treatment targeting all adults/adults with underlying diseases only scenarios resulted in a greater number of ICU admissions relative to standard care since the number of months when ICU capacity is not exceeded is lower for the treatment scenarios compared to the standard care scenario.

5 Negative indicates a reduced demand for admission based on the treatment efficacy for reducing the severity rate, and positive indicates an increased demand for admission based on the treatment efficacy for reducing recovery time during months when the hospital/ICU capacity is exceeded.

6 Negative indicates the cost per admission allowed under the increased demand for admissions with a high epidemic surge, and positive indicates the cost per prevented admission under the decreased demand for admission with a suppressed epidemic curve. The treatment efficacy for reducing the admission rate can reduce the total number of admissions, but the treatment efficacy for reducing recovery time enables more severe patient admissions when the ICU capacity is exceeded during a high epidemic surge.

## SUPPLEMENTARY MATERIALS

Supplementary Material 1. Schematic representation of the modeling approach

Supplementary Material 2. Model input parameters

Supplementary Material 3. Model projection for treatment to all adult patients scenario

Supplementary Material 4. Health outcome, costs and ICERs by scenarios

References
