# Supplementary material for: Model-based cost-effectiveness analysis of oral antivirals against SARS-CoV-2 in Korea
Source: Epidemiol Health. 2022 Mar 12;44:e2022034. doi: 10.4178/epih.e2022034 (PMC9350420; doi:10.4178/epih.e2022034)
Supplement: Supplementary Material 1. — Schematic representation of the modeling approach [file epih-44-e2022034-suppl1.docx]

**Supplementary Material 1. Schematic representation of the modeling approach**

We adapted an age-specific, deterministic compartmental model to capture the epidemiological dynamics of SARS-CoV-2 and the situation facing COVID-19 control decision-making in the Republic of Korea during the pandemic (1 July 2021-21 November 2021) and 13 months into the future (22 November 2021 – 31 December 2022). For this study, we used the estimated model outputs between January 2022 -December 2022**.[1]** The model comprised twelve disease-related states: uninfected, exposed, asymptomatic, true positive, symptomatic, hospitalized, recovered, dead, and vaccinated. (A. Model) In this model, both the vaccinated and unvaccinated susceptible populations may be exposed and develop COVID-19 infection, which becomes detected by mass screening/testing and develop symptoms. Infected populations include asymptomatic (undetected/test positive) and symptomatic (detected) populations, and we assumed those who are test positive to be isolated and not to contribute to the transmission. We considered the natural recovery of the infected population and progression from asymptomatic to symptomatic groups. The symptomatic population will be either recovered or COVID-19 related dead. The population was subdivided into four groups based on age: 0-19 years, 20-39 years, 40-59 years, 60 years and above, and assumed age-specific contact patterns based on the published reference. (B. Age contact matrix) For each target scenario (all adult patients, elderly patients, adult patients with underlying diseases), we changed the rate at which symptomatic individuals being admitted to hospital (α) with different efficacy levels by treatment scenarios.

| 1. Model | 1. Age contact matrix |
| --- | --- |
| 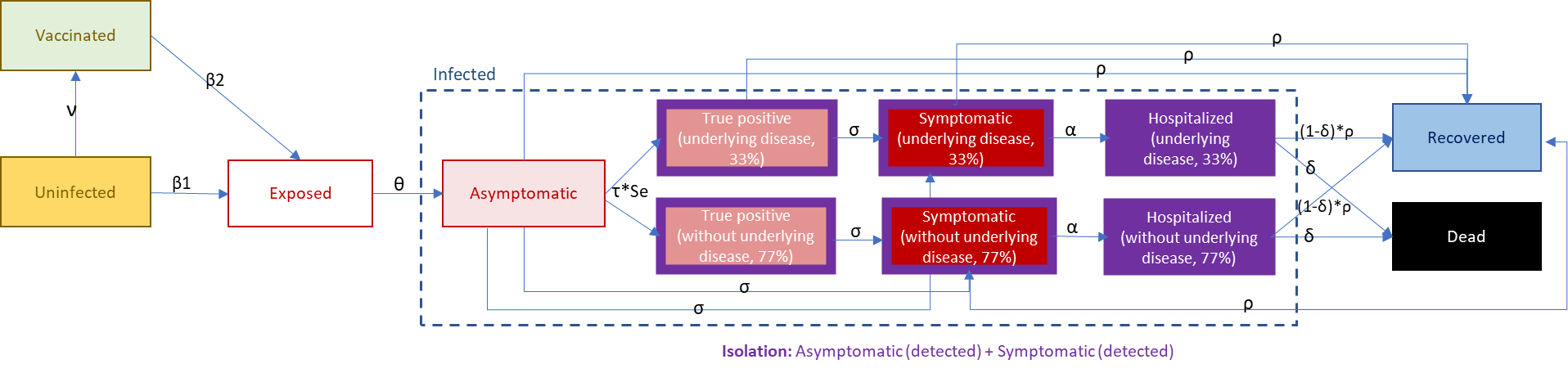 |  |

| **Model parameters** | |
| --- | --- |
| V | Uninfected, vaccinated individuals |
| U | Uninfected, susceptible individuals |
| E | Exposed, asymptomatic, non-infectious |
| A | Infected, asymptomatic non-infectious and non-symptomatic (test negative, if screened) |
| TP | Infected, asymptomatic, (true) positive test result 🡪 isolated from the active transmission |
| S | Infected, symptomatic (true) positive test result 🡪 isolated from the active transmission |
| R | Recovered (assuming immune for 1 year; no role in the system) |
| D | Dead |
| β | rate at which infected individuals contact susceptible and infect them |
| α | rate at which symptomatic individuals being admitted to hospital |
| τ | rate at which individuals being screened and tested |
| θ | rate at which exposed individuals advance to the asymptomatic, infectious compartment |
| δ | rate at which individuals in the symptomatic compartment die |
| ρ | rate at which infected individuals recover from disease and are removed |
| σ | rate of symptom onset for infected individuals |
| Se | sensitivity of the screening test |
| ν | rate at which individuals being vaccinated |
