# Supplementary material for: Model-based cost-effectiveness analysis of oral antivirals against SARS-CoV-2 in Korea
Source: Epidemiol Health. 2022 Mar 12;44:e2022034. doi: 10.4178/epih.e2022034 (PMC9350420; doi:10.4178/epih.e2022034)
Supplement: Supplementary Material 2. — Model input parameters [file epih-44-e2022034-suppl2.docx]

**Supplementary Material 2. Model input parameters**

| **Population as of July 1 2021** | | **Age 0-19** | **Age 20-39** | **Age 40-59** | **Age 60+** | **Reference** |
| --- | --- | --- | --- | --- | --- | --- |
| Total population (N) | | 8,809,740 | 13,991,940 | 17,101,260 | 11,919,060 | KDCA (total population =51,822,000) |
| Uninfected (U) | | 8,808,740 | 13,990,940 | 15,096,260 | 8,917,060 | Total population-Infected-vaccinated-recovered-dead |
| Vaccinated (V) | | 724 | 1,257,656 | 1,360,168 | 7,290,718 | KDCA by June 30, 2021 |
| Exposed (E) | | 1,000 | 2,000 | 2,000 | 2,000 | Assumed by incidence level |
| Asymptomatic (A) | | 1,000 | 2,000 | 2,000 | 2,000 |  |
| Test positive (TP) | | 100 | 200 | 400 | 300 | KDCA |
| Symptomatic (S) | | 100 | 200 | 400 | 300 | KDCA |
| Recovered (R) | | 50,000 | 50,000 | 50,000 | 100,000 | KDCA & assumptions: cumulative confirmed cases + natural recovery since March 2020 |
| Dead (D) | | 0 | 0 | 500 | 1,000 | KDCA |
| **Model parameters** | | **Age 0-19** | **Age 20-39** | **Age 40-59** | **Age 60+** |  |
| Contact matrix | Age 0-19 | 4 | 3 | 2 | 1 | [2-4] & assumptions |
|  | Age 20-39 | 3 | 3 | 2 | 1 |  |
|  | Age 40-59 | 2 | 2 | 3 | 1 |  |
|  | Age 60+ | 1 | 1 | 1 | 1 |  |
| Effective vaccinations per day^§^(%) (ν) | Jul 1-Nov 20, 2021 | 9,787  (0.1%) | 53,998  (0.4%) | 73,070  (0.4%) | 29,964  (0.3%) | The average number of vaccinations per day by age group from observed data |
|  | Nov 21-Dec 30, 2021 | 29,361  (0.3%) | 26999  (0.2%) | 36,335  (0.2%) | 2,996  (0.3%) | Assumed age specific vaccination uptake by June 2022 to reach 70% effective coverage (+/- 20% were assumed for variation for sensitivity analyses) |
|  | Jan 1-Jun 30, 2022 | 19,574  (0.2%) | 16199  (0.1%) | 14,614  (0.01%) | 300  (0.0%) |  |
|  | Jul 1-Dec 30, 2022 | 1957  (0.0%) | 1079  (0.0%) | 1461  (0.0%) | 60  (0.0%) |  |
| Progression rate from asymptomatic to symptomatic status | | 0.14 | 0.2 | 0.15 | 0.12 | Probability of symptoms given infection: 66% (0-19 age) 74% (20-39 age) 68% (40-59 age) 62% (60 age over) from KDCA data &  Estimated daily rate by the formula:  Probability of symptoms given infection *recovery rate/(1-recovery rate) |
| Hospital admission rate among symptomatic patients (α) | | 0.00002 | 0.00152 | 0.00739 | 0.07939 | A weighted average of the probability of severity progression rate between unvaccinated and vaccinated populations from KDCA data. |
| Symptomatic case fatality risk (δ) | | 0.00004 | 0.00001 | 0.00098 | 0.047 | A weighted average of the probability of case fatality rate between unvaccinated and vaccinated populations from KDCA data. |
| Vaccine effectiveness against infection | 1^st^ dose AstraZeneca | 50% (30%) | | | | [5] & assumptions: The efficacy was multiplied to respective age group along with the respective proportion of delta variants among tested individuals |
|  | 1^st^ dose Johnson & Johnson | 50% (30%) | | | |  |
|  | 1^st^ dose Pfizer/Moderna | 50% (35%) | | | |  |
|  | 2^nd^ dose AstraZeneca | 77% (66%) | | | |  |
|  | 2^nd^ dose Pfizer/Moderna | 95% (88%) | | | |  |
|  | 3^rd^ dose AstraZeneca | 80% (70%) | | | |  |
|  | 3^rd^ dose Pfizer/Moderna | 98% (90%) | | | |  |
| Transmissibility (β) | Jul 1-Oct 15, 2021 | 0.05 | | | | Estimated by EpiEstim based on serial interval distribution and case reporting data |
|  | Oct 16-Nov 21, 2021 | 0.08 | | | |  |
|  | Nov 21 2021- Dec 30, 2022 | 0.16 | | | | Taken by the assumption of 3 times greater transmissibility of the new variants from the average Rt estimate between July 1 to Nov 21 2021 (basecase) [6] |
| Social distancing control (Nov 21 2021-Feb 30 2022) | Weak control | general 40% reduction in transmission | | | | [7]& assumptions |
|  | Moderate control | general 50% reduction in transmission | | | |  |
|  | Strong control | general 60% reduction in transmission | | | |  |
| Testing intensity among asymptomatic patients (Jul 1 2021 to Dec 30, 2022) (τ) | | 0.14 | | | | Estimated daily testing rate among asymptomatic patients based on the observed total daily testing volume and the proportion of testing of exposed and asymptomatic individuals (assumed as 30% from seropositive prevalence data); Daily testing rate (14%) = average dailiy testing volume (35,000)/ total suspected individuals including uninfected, exposed and asymptomatic populations (assumed as 250,000) (+/- 20% were assumed for variation for sensitivity analyses during Nov 21 2021 to Dec 30 2022) |
| Incubation rate (θ) | | 0.33 | | | | 3-4 days [8] ; daily rate estimated as 1/3 |
| Diagnostic sensitivity (Se) | | 0.95 | | | | Assumption |
| Recovery rate (ρ) | | 0.07 | | | | 14 days [9] ; daily rate estimated as 1/14 |

KDCA, Korea Disease Control and Prevention Agency

^§^ Vaccination rates differ by age group by the government strategies and population size. The government prioritized vaccination to 60+ years age group, incrementally expanded access to 40-59, 20-39, and 0-19 years age groups.
