# Supplementary material for: Model-based cost-effectiveness analysis of oral antivirals against SARS-CoV-2 in Korea
Source: Epidemiol Health. 2022 Mar 12;44:e2022034. doi: 10.4178/epih.e2022034 (PMC9350420; doi:10.4178/epih.e2022034)
Supplement: Supplementary Material 3. — Model projection for treatment to all adult patients scenario [file epih-44-e2022034-suppl3.docx]

**Supplementary Material 3. Model projection for treatment to all adult patients scenario**

**Model outputs:** From an existing COVID 19 epidemiology model, [1] we projected the peak hospital/ intensive care unit (ICU) beds based on hospital/ICU prevalence and the number of people requiring hospital/ICU admissions based on hospital/ICU incidence by respective scenarios: (i) status quo, (ii) administering molnupiravir to all adults symptomatic patients, (iii) administering Nirmatrelvir/Ritonavir to all adults symptomatic patients between January and December 2022 in the Republic of Korea. We assumed using treatment (Molnupiravir or Nirmatrelvir/Ritonavir) may reduce the admission rate of the specific target group by molnupiravir (30% efficacy) or nirmatrelvir/ritonavir (87% efficacy) compared to standard care. We also assumed these treatments reduce recovery time 4 days in hospital/ICU (from average 13 to 9 days) and thus allow more patients to be admitted during the months of hospital/ICU capacity are breached. We used peak hospital/ICU beds (by month) estimates to identify peak capacity months (“1” if hospital/ICU capacity is breached; “0” if hospital/ICU capacity is not breached). We then used the peak capacity months and the total number of people requiring hospital/ICU admissions to identify the total number of hospital/ICU admissions during peak months and non-peak months (same as the original estimate of number of people requiring hospital/ICU admissions).

**Health outcomes:** We estimated number of severe patients averted and number of net admission allowed/averted by each treatment strategy to respective target group relative to the standard care in three aspects: 1) number of severe patients averted by treatment efficacy to reduce severity rate (30% for Molnupiravir and 87% for Nirmatrelvir/Ritonavir) relative to standard care; 2) number of admission averted by treatment efficacy reduce severity rate (30% for Molnupiravir and 87% for Nirmatrelvir/Ritonavir) relative to standard care; and 3) number of admission allowed by treatment efficacy to reduce recovery time in hospital/ICU relative to standard care. The month of hospital/ICU capacity is breached is determined by the peak of the hospital/ICU prevalence of each month and the number of hospital beds (25000) and ICU beds (1500) respectively.

- First, the number of severe patients averted in all months was calculated by “Number of people who need hospital/ICU admission at status quo” subtracted by “Number of people who need hospital/ICU admission with treatment” regardless of the capacity limit.
- Similarly, the number of admission averted when capacity is not breached was calculated by the number of total admission at status quo subtracted number of total admission with treatment. Total number of admission is as same as number of people who need hospital/ICU admission when hospital/ICU capacity is not breached but total number of admission is different (as described below) when hospital/ICU capacity is breached.
- Finally, the number of admission allowed when capacity is breached was calculated by a number of total admission at status quo subtracted the number of total admission with treatment. (i) number of total admission at status quo is calculated by the maximum number of people treated if the average number of hospital/ICU days is in a month (13 days with standard care) multiplied by the number of months that capacity is breached; (ii) number of total admission at status quo is calculated by the maximum number of people treated if the average number of hospital/ICU days is in a month (9 days with treatment) multiplied by the number of months that capacity is breached.

**Costs:** We estimated costs in three aspects: 1) Health system costs for the months with treatment in peak months and non-peak months; 2) Health system costs for the months without treatment (status quo) in peak months and non-peak months; 3) treatment cost. During the peak months, health systems costs were calculated by the total number of admission (83,333 in hospital and 5,000 in ICU per month with treatment due to recovery time benefit; 57,692 in hospital and 3,462 in ICU per month without treatment) multiplied by hospital/ICU cost per person; During the non-peak months, health systems costs were calculated by the total number of admission (the number of people who need the hospital/ICU admission) multiplied by hospital/ICU cost per person.

Model analytic approach is similar to other target groups (elderly patients and adult patients with underlying diseases).

| **2. Health Systems Capacity (total admission)** | | | |  |
| --- | --- | --- | --- | --- |
| Level | Number of beds | Total people treated if average number of inpatient days is 13 in a month (assuming 30 day month) | Total number of people treated if average number of inpatient days is 9 in a month (assuming 30 day month) by mol | Additional Patients in ICU due to treatment per month |
| Hospital | 25,000 | 57,692 | 83,333 | 25,641 |
| ICU | 1,500 | 3,462 | 5,000 | 1,538 |

| 1. **Health Outcome** | | | | | | | | |
| --- | --- | --- | --- | --- | --- | --- | --- | --- |
| Drug | Number of people who need hospital admission | Number of people who need ICU admission | Averted number of people who need hospital/ICU admission | Number of patients getting hospital care when capacity is breached | Number of patients getting ICU care when capacity is breached | Hospital admission for all months when capacity is not exceeded | ICU admission for all months when capacity is not exceeded | Additional patients receiving hospital/ICU care if all patients receive treatment |
| Status quo | 181,931 | 54,579 | NA | 115,385 | 13,846 | 68,873 | 6,476 | NA |
| Molnupiravir | 135,803 | 40,740 | -59,967 | 0 | 20,000 | 135,803 | 4,772 | 44,005 |
| Nirmatrelvir/Ritonavir | 36,949 | 11,083 | -188,478 | 0 | 10,000 | 36,949 | 4,080 | 153,551 |

| **4. Cost** |  |  |  |
| --- | --- | --- | --- |
| Drug | Drug cost | Hospital operation costs | ICU operation costs |
| Status quo | NA | $49,196,783 | $16,765,777 |
| Molnupiravir | $1,717,867,200 | $36,259,401 | $20,436,900 |
| Nirmatrelvir/ritonavir | $1,717,867,200 | $9,865,383 | $11,616,000 |
