# Supplementary material for: Model-based cost-effectiveness analysis of oral antivirals against SARS-CoV-2 in Korea
Source: Epidemiol Health. 2022 Mar 12;44:e2022034. doi: 10.4178/epih.e2022034 (PMC9350420; doi:10.4178/epih.e2022034)
Supplement: Supplementary Material 4. — Health outcome, costs and ICERs by scenarios [file epih-44-e2022034-suppl4.docx]

**Supplementary Material 4. Health outcome, costs and ICERs by scenarios**

|  | **Standard care (without treatment)** | Treatment targeting all adult patients | Treatment targeting elderly patients only | Treatment targeting adult patients with underlying disease only | Treatment targeting all adult patients | Treatment targeting elderly patients only | Treatment targeting adult patients with underlying disease only | Treatment targeting all adult patients | Treatment administered elderly patients | Treatment targeting adult patients with underlying disease only |
| --- | --- | --- | --- | --- | --- | --- | --- | --- | --- | --- |
|  |  | **Reduced admission efficacy 10% scenario** | | | Reduced admission efficacy 30% **scenario** | | | Reduced admission efficacy 50% **scenario** | | |
| **Health outcome by intervention scenarios ^a^** | | | | | | | | | | |
| Number of severe patients who require hospital admission ^b^ (A) | 181,931 | 167,041 | 179,742 | 177,464 | 135,803 | 174,517 | 168,088 | 102,187 | 167,817 | 158,007 |
| Number of severe patients who require ICU admission (B) | 54,579 | 50,113 | 53,921 | 53,240 | 40,740 | 52,354 | 50,425 | 30,657 | 50,346 | 47,401 |
| Total number of severe patients who require hospital/ICU admission (C) | 236,510 | 217,154 | 233,663 | 230,704 | 176,543 | 226,871 | 218,513 | 132,844 | 218,163 | 205,408 |
| Total prevented severe cases (D) |  | -19,356 | -2,847 | -5,806 | -59,967 | -9,639 | -17,997 | -103,666 | -18,347 | -31,102 |
| Number of patients receiving hospital care during months when capacity is exceeded (E) | 115,385 | 166,667 | 166,667 | 166,667 | 0 ^c^ | 166,667 | 166,667 | 0 ^c^ | 166,667 | 83,333 |
| Number of patients receiving ICU care during months when capacity is exceeded (F) | 13,846 | 20,000 | 20,000 | 20,000 | 20,000 | 20,000 | 20,000 | 20,000 | 20,000 | 20,000 |
| Hospital admission during months when capacity is not exceeded ^b^ (G) | 68,873 | 63,040 | 68,128 | 67,121 | 135,803 | 66,380 | 63,480 | 102,187 | 64,235 | 91,419 |
| ICU admission during months when capacity is not exceeded ^d^ (H) | 6,476 | 5,924 | 6,429 | 6,310 | 4,772 | 6,316 | 5,965 | 3,556 | 6,181 | 5,600 |
| Total admissions under the current health system capacity (I: E+F+G+H) | 204,580 | 255,631 | 261,224 | 260,098 | 160,575 | 259,363 | 256,112 | 125,743 | 257,083 | 200,352 |
| Net total hospital/ICU admission by treatment under the current health system capacity ^e^ (J) |  | 51,051 | 56,644 | 55,518 | -44,005 | 54,783 | 51,532 | -78,837 | 52,503 | -4,227 |
| **Cost (million USD)** | | | | | | | | | | |
| Drug costs (K) | NA | $1,718M | $58M | $515M | $1,718M | $58M | $515M | $1,718M | $58M | $515M |
| Hospital costs (L) | $49M | $61M | $63M | $62M | $36M | $62M | $61M | $27M | $62M | $47M |
| ICU costs (M) | $17M | $21M | $22M | $22M | $20M | $22M | $21M | $19M | $22M | $21M |
| Total costs (N) | $66M | $1,801M | $143M | $599M | $1,775M | $142M | $598M | $1,765M | $142M | $583M |
| **Incremental costs (O)** |  | **$1,735M** | **$77M** | **$534M** | **$1,707M** | **$76M** | **$532M** | **$1,699M** | **$76M** | **$517M** |
| **ICER** | | | | | | | | | | |
| **ICER: Cost per prevented severe case(D/O)** |  | **$89,617** | **$26,994** | **$91,891** | **$28,492** | **$7,915** | **$29,575** | **$16,386** | **$4,121** | **$16,628** |
| **ICER: Cost per admission/prevented admission ^f^ (J/O))** |  | **-$33,978** | **-$1,357** | **-$9,610** | **$38,828** | **-$1,393** | **-$10,329** | **$21,546** | **-$1,440** | **$122,336** |
|  |  | **Reduced admission efficacy 70% scenario** | | | **Reduced admission efficacy 87% scenario** | | | **Reduced admission efficacy 90% scenario** | | |
| **Effectiveness** | | | | | | | | | | |
| Number of severe patients who require hospital admission ^b^ (A) |  | 65,358 | 158,823 | 146,956 | 36,949 | 150,506 | 138,433 | 23,899 | 146,202 | 134,520 |
| Number of severe patients who require ICU admission (B) |  | 19,608 | 47,646 | 44,087 | 11,083 | 45,152 | 41,530 | 7,169 | 43,860 | 40,356 |
| Total number of severe patients who require hospital/ICU admission (C) |  | 84,966 | 206,469 | 191,043 | 48,032 | 195,658 | 179,963 | 31,068 | 190,062 | 174,876 |
| Total prevented severe cases (D) |  | -151,544 | -30,041 | -45,467 | -188,478 | -40,852 | -56,547 | -205,442 | -46,448 | -61,634 |
| Number of patients receiving hospital care during months when capacity is exceeded (E) |  | 0 | 166,667 | 0 | 0 | 83,333 | 0 | 0 | 0 | 0 |
| Number of patients receiving ICU care during months when capacity is exceeded (F) |  | 15,000 | 20,000 | 20,000 | 10,000 | 20,000 | 20,000 | 0 | 20,000 | 20,000 |
| Hospital admission during months when capacity is not exceeded ^b^ (G) |  | 65,358 | 61,419 | 146,956 | 36,949 | 88,009 | 138,433 | 23,899 | 85,509 | 134,520 |
| ICU admission during months when capacity is not exceeded ^d^ (H) |  | 3,186 | 5,993 | 5,211 | 4,080 | 5,817 | 4,913 | 7,169 | 5,720 | 4,779 |
| Total admissions under the current health system capacity (I: E+F+G+H) |  | 83,544 | 254,079 | 172,167 | 51,029 | 197,159 | 163,346 | 31,068 | 111,229 | 159,299 |
| Net total hospital/ICU admission by treatment under the current health system capacity ^e^ (J) |  | -121,036 | 49,499 | -32,413 | -153,551 | -7,420 | -41,234 | -173,512 | -93,351 | -45,281 |
| **Cost (million USD)** | | | | | | | | | | |
| Drug costs (K) |  | $1,718M | $58M | $515M | $1,718M | $58M | $515M | $1,718M | $58M | $515M |
| Hospital costs (L) |  | $17M | $61M | $39M | $10M | $46M | $37M | $6M | $45M | $36M |
| ICU costs (M) |  | $15M | $21M | $21M | $12M | $21M | $21M | $6M | $21M | $20M |
| Total costs (N) |  | $1,750M | $141M | $575M | $1,739M | $125M | $573M | $1,730M | $125M | $572M |
| **Incremental costs (O)** |  | **$1,684M** | **$75M** | **$509M** | **$1,673M** | **$59M** | **$507M** | **$1,664M** | **$59M** | **$506M** |
| **ICER** | | | | | | | | | | |
| **ICER: Cost per prevented severe case(D/O)** |  | **$11,115** | **$2,487** | **$11,204** | **$8,878** | **$1,454** | **$8,964** | **$8,101** | **$1,263** | **$8,206** |
| **ICER: Cost per admission/prevented admission ^f^ (J/O)** |  | **$13,916** | **-$1,509** | **$15,717** | **$10,898** | **$8,006** | **$12,293** | **$9,591** | **$628** | **$11,169** |

COVID-19, coronavirus disease 2019; ICER, incremental cost-effectiveness ratio; ICU, intensive care unit;

a. The health outcome is the total population impact based on the epidemiology model targeting each respective patient group.

b. “Hospital admissions during months when capacity is not exceeded (G)” is the same as “Number of the population who require hospital admission (A)” for targeting all adult patients since the hospital capacity is never exceeded in all months of 2022.

c. 0 since treatment targeting all adults patients can suppress the epidemic curve below the ICU capacity limit for all months.

d. Treatment targeting all adults/adults with underlying diseases only scenarios resulted in a greater number of ICU admissions relative to standard care since the number of months when ICU capacity is not exceeded is lower for the treatment scenarios compared to the standard care scenario.

e. Negative indicates a reduced demand for admission based on the treatment efficacy for reducing the severity rate, and positive indicates an increased demand for admission based on the treatment efficacy for reducing recovery time during months when the hospital/ICU capacity is exceeded.

f. Negative indicates the cost per admission allowed under the increased demand for admissions with a high epidemic surge, and positive indicates the cost per prevented admission under the decreased demand for admission with a suppressed epidemic curve.
