# Supplementary material for: Model-based cost-effectiveness analysis of oral antivirals against SARS-CoV-2 in Korea
Source: Epidemiol Health. 2022 Mar 12;44:e2022034. doi: 10.4178/epih.e2022034 (PMC9350420; doi:10.4178/epih.e2022034)
Supplement: References [file epih-44-e2022034-suppl5.docx]

References

1. Jo Y, Shrestha S, Radnaabaatar M, Park H, Jung J. Optimal social distancing policy for COVID-19 control in South Korea: a model-based analysis. 2022. Submitted.

2. Mossong J, Hens N, Jit M, Beutels P, Auranen K, Mikolajczyk R, et al. Social contacts and mixing patterns relevant to the spread of infectious diseases. PLoS Med. 2008;5(3):e74. doi: 10.1371/journal.pmed.0050074.

3. Prem K, Cook AR, Jit M. Projecting social contact matrices in 152 countries using contact surveys and demographic data. PLoS Comput Biol. 2017;13(9):e1005697. doi: 10.1371/journal.pcbi.1005697.

4. Arregui S, Aleta A, Sanz J, Moreno Y. Projecting social contact matrices to different demographic structures. PLoS Comput Biol. 2018;14(12):e1006638. doi: 10.1371/journal.pcbi.1006638.

5. Baden LR, El Sahly HM, Essink B, Kotloff K, Frey S, Novak R,et al. Efficacy and Safety of the mRNA-1273 SARS-CoV-2 Vaccine. N. Engl. J. Med. 2021;384(5):403-416. doi: 10.1056/NEJMoa2035389.

6. Liu Y, Rocklov J. The reproductive number of the Delta variant of SARS-CoV-2 is far higher compared to the ancestral SARS-CoV-2 virus. J Travel Med. 2021;28(7). doi: 10.1093/jtm/taab124.

7. Rost G, Bartha FA, Bogya N, Boldog P, Denes A, Ferenci T, et al. Early Phase of the COVID-19 Outbreak in Hungary and Post-Lockdown Scenarios. Viruses. 2020;12(7). doi: 10.3390/v12070708.

8. He X, Lau EHY, Wu P, Deng X, Wang J, Hao X, et al. Temporal dynamics in viral shedding and transmissibility of COVID-19. Nat Med. 2020;26(5):672-5. doi: 10.1038/s41591-020-0869-5.

9. Lauer SA, Grantz KH, Bi Q, Jones FK, Zheng Q, Meredith HR, et al. The Incubation Period of Coronavirus Disease 2019 (COVID-19) From Publicly Reported Confirmed Cases: Estimation and Application. Ann Intern Med. 2020;172(9):577-82. doi: 10.7326/M20-0504.
